# Perhexiline Therapy in Patients with Type 2 Diabetes: Incremental Insulin Resistance despite Potentiation of Nitric Oxide Signaling

**DOI:** 10.3390/biomedicines10102381

**Published:** 2022-09-23

**Authors:** Cher-Rin Chong, Saifei Liu, Hasan Imam, Tamila Heresztyn, Benedetta C. Sallustio, Yuliy Y. Chirkov, John D. Horowitz

**Affiliations:** 1 Adelaide Medical School, The University of Adelaide, Adelaide 5000, Australia; 2 Basil Hetzel Institute for Translational Health Research, The Queen Elizabeth Hospital, Woodville South 5011, Australia; 3 School of Medical Sciences, The University of Adelaide, Adelaide 5000, Australia

**Keywords:** perhexiline, type 2 diabetes, nitric oxide, insulin resistance

## Abstract

Perhexiline (Px) inhibits carnitine palmitoyltransferase 1 (CPT1), which controls uptake of long chain fatty acids into mitochondria. However, occasional cases of hypoglycaemia have been reported in Px-treated patients, raising the possibility that Px may also increase sensitivity to insulin. Furthermore, Px increases anti-aggregatory responses to nitric oxide (NO), an effect which may theoretically parallel insulin sensitization. We therefore sought to examine these relationships in patients with stable Type 2 diabetes (T2D) and cardiovascular disease (n = 30). Px was initiated, and dosage was titrated, to reach the therapeutic range and thus prevent toxicity. Investigations were performed before and after 2 weeks, to examine changes in insulin sensitivity and, utilizing aggregometry in whole blood, platelet responsiveness to the anti-aggregatory effects of the NO donor sodium nitroprusside (SNP). Other parameters that affect may affect NO signalling were also evaluated. Px substantially potentiated inhibition of platelet aggregation by SNP (from 16.7 ± 3.0 to 27.3 ± 3.7%; *p* = 0.005). Px did not change fasting blood glucose concentrations but reduced insulin sensitivity (HOMA-IR score increased from median of 4.47 to 6.08; *p* = 0.028), and increased fasting plasma insulin concentrations (median 16.5 to 19.0 mU/L; *p* = 0.014). Increases in SNP responses tended (r = −0.30; *p* = 0.11) to be reciprocally related to increases in HOMA-IR, and increases in HOMA-IR were greater (*p* = 0.002) in patients without NO-sensitizing effects. No patient developed symptomatic hypoglycaemia, nor was there any other short-term toxicity of Px. Thus, in patients with stable T2D and cardiovascular disease, Px increases anti-aggregatory responsiveness to NO, but is not an insulin sensitizer, and does not induce hypoglycaemia. Absence of NO-sensitizing effect occurs in approximately 30% of Px-treated patients with T2D, and is associated with induction of insulin resistance in these patients.

## 1. Introduction

Type 2 diabetes (T2D) is an increasingly prevalent problem throughout the world and is associated with a substantial increase in prevalence of both stable myocardial ischaemia, infarction, heart failure, and associated mortality risk. Furthermore, patients with T2D are at increased risk for carcinogenesis [1]. 

These adverse prognostic aspects of T2D have contributed to investigations to identify biochemical modulators of cardiovascular risk, as well of the nexus between impaired responsiveness to insulin and propensity towards both myocardial ischaemia and development of cancer. It has been shown that extents of both insulin resistance [2] and of hyperglycaemia, especially at times of clinical crises [3], represent adverse prognostic markers for cardiovascular outcomes. Furthermore, severe hyperglycaemia represents a basis for increased mitochondrial formation of superoxide anion (O_2_^−^), a major mediator of many of the cardiovascular complications of diabetes [4], with resultant “scavenging” of nitric oxide (NO) and therefore impairment of its vasodilator and anti-aggregatory effects, known as “NO resistance”. NO resistance represents an adverse prognostic marker [5], whether measured via the coronary vasodilator [6], or the anti-aggregatory effects of NO [5]. While NO resistance primarily reflects the impact of oxidative stress on “scavenging” of NO [7] and activity of the “receptor” for NO, soluble guanylate cyclase [8], a number of other factors may also modulate integrity of NO signalling. These include asymmetric dimethylarginine (ADMA), an endogenous inhibitor of NO synthases [9], myeloperoxidase (MPO), which is released from activated neutrophils and inhibits the metabolic clearance of ADMA [10], and thrombospondin-1 (TSP-1), which is released from platelet alpha granules and blocks NO signalling, thus prediposing to platelet aggregation [11].

Insulin infusion [7], the ACE inhibitor ramipril [12], the hydrogen sulphide donor N-acetylcysteine [13] and the prophylactic anti-anginal agent perhexiline (Px) [14] have all been shown to attenuate NO resistance, although the precise mechanism(s) underlying this beneficial effect have never been fully defined. 

In the case of Px, both its impact on insulin sensitivity in patients with diabetes and its effects on maintenance of homeostasis at the platelet level are issues of increasing importance. The range of clinical utility of Px has expanded considerably, following demonstration that its potential long-term hepatotoxicity and neurotoxicity can be prevented by maintenance of plasma Px concentrations within a defined therapeutic range [15,16] and that Px is safe for patients with cardiac and renal insufficiency [17]. 

Px is now recognized as inducing a “metabolic” prophylactic antianginal effect, with a major mechanism of action identified as induction of a cardiac metabolic shift from long-chain fatty acid to glucose oxidation via inhibition of the rate-limiting enzyme carnitine palmitoyltransferase-1 (CPT-1) and, to a lesser extent, CPT-2 [18]. Therefore, in theory, Px should activate a “Randle shift” [19], whereby there is a compensatory increase in glucose utilization when fatty acid utilization is suppressed. In theory, this adjustment of substrate utilization would lead to an increase in efficiency of cardiac oxygen utilization [18,20,21]. These effects have opened up new therapeutic options for Px, which include the management of systolic heart failure [17,21], and non-obstructive hypertrophic cardiomyopathy [22]. 

Recently, several preclinical studies have suggested that Px also exerts substantial antineoplastic effects, both in tumour cell lines and in vivo [23,24,25,26,27]. The concept of an agent with combined antineoplastic and cardioprotective effects is very attractive on a theoretical basis. However, to date, there has been little evidence that effecting a Randle shift in cardiac metabolism, representing a means for maintaining cellular energetics despite reduced substrate availability, might interact directly either with the actions of insulin (in promoting cellular uptake of glucose), or the Warburg effect (of activating anaerobic metabolism in association with inappropriate cellular proliferation) [28]. 

The introduction of Px into the therapeutic arena preceded the utilization of therapeutic drug monitoring. Several cases of hypoglycaemia, sometimes severe, were reported in the early literature [29,30,31,32]. However, no detailed studies of Px effects on insulin signaling have been reported to date. The currently reported study was therefore undertaken to evaluate the effects of Px therapy on insulin responsiveness in patients with stable T2D and cardiovascular disease, and to determine whether its effects on insulin signaling might parallel changes in tissue responsiveness to NO. 

## 2. Materials and Methods

### 2.1. Patient Selection

Adult patients with stable T2D were considered for inclusion if they were concurrently under consideration for the initiation of Px treatment for the management of cardiovascular disease states including refractory angina pectoris, systolic heart failure, or symptomatic aortic valve stenosis [33]. Exclusion criteria were (1) current or potential pregnancy, (2) concurrent therapy with any P2Y_12_ receptor antagonist (which would obscure effects on planned platelet aggregation studies), or (3) previous adverse effect of Px. 

### 2.2. Study Design

The study was designed as a comparison of the effects of two weeks’ treatment with Px on (i) insulin sensitivity in patients with well-controlled Type 2 diabetes mellitus (primary endpoint), and (ii) platelet responsiveness to the anti-aggregatory effects of NO. Stability of diabetic control was characterized by no recent emergency treatment of diabetes or adjustment of hypoglycaemic medications. Plasma concentrations of HbA1c were less than 9%. Plasma concentrations of ADMA, TSP-1 and of MPO were also measured because of because of their potential roles as modulators of NO effect. TXNIP expression in platelets was also measured because of its previously demonstrated reciprocal relationship with tissue responsiveness to NO [34]. 

Following baseline evaluations, Px treatment was initiated with a rapid loading regimen of 600 mg on the first day, followed by adjustment of dosage on the basis of initial plasma concentrations of Px and its monohydroxylated metabolite [35]. Plasma Px concentrations were re-assayed after 2 weeks’ of treatment. 

### 2.3. Investigations 

The following were performed before initiation of Px, and at the end of the study period. Patients were advised to fast overnight, and blood samples were drawn into acid citrate anticoagulant, on the following morning between 0800 and 0900 h. Investigations performed included:

1. Determination of fasting blood glucose levels and plasma insulin concentrations to measure insulin resistance as HOMA-IR, representing the primary endpoint, and insulin sensitivity by QUICKI score.
2. Measurement of platelet pro-aggregatory responses to ADP and anti-aggregatory responses to the NO donor sodium nitroprusside (SNP). Whole blood impedance aggregometry (Model 560, Chrono-log^®^, Haverstown, PA, USA) was used to record platelet aggregation, in Ohms [36]. Blood samples were stirred at 900 rpm at 37 °C, and platelet aggregation was induced by 2.5 μM ADP; inhibition of aggregation was induced by 10 μM of SNP.
3. Plasma concentrations of TSP-1 [37] were assayed with ELISA kit (R&D systems^®^, Minneapolis, MN, USA); ADMA was assayed using a previously published HPLC assay [38,39]; and MPO using an ELISA kit (Mercodia^®^, Uppsala, Sweden) [40]. Platelet content of TXNIP was also determined by immunohistochemistry [34,41].

### 2.4. Statistical Methodology

The study results were assessed based on intention-to-treat principles and the limit of statistical significance was taken as *p* < 0.05 using GraphPad Prism (version 9, San Diego, CA, USA). All parameters were compared on a paired basis before and after 2 weeks’ Px therapy, using either Student’s paired t-test or a paired Wilcoxon test as appropriate. The inclusion of 30 patients ensured a power of the primary endpoint (insulin resistance measured by HOMA-IR) of α = 0.05, β = 0.80 to detect a 0.5 SD fluctuation post Px. Correlations between Px effects on HOMA-IR and SNP response were sought using Pearson’s correlation coefficient. Data are expressed as mean ± SEM unless otherwise stated. 

## 3. Results

Clinical data related to the participants are summarized in Table 1. In general, this was an ageing group of patients with well-controlled T2D and mild to moderate renal impairment. The most common indication for Px therapy was angina pectoris refractory to other anti-anginal agent. These patients were still symptomatic prior to initiation of Px therapy despite receiving at least one long-acting prophylactic anti-anginal agent. Other indications for Px therapy included symptomatic heart failure [20,21] and aortic stenosis [42]. Most patients were also receiving either ACE inhibitors or angiotensin receptor blocker therapy. Regarding treatment for T2D, most patients received more than one therapy, and metformin remained the most utilized oral hypoglycaemic agent.

Thirty-three patients provided informed consent. One patient developed nausea and withdrew from the study, and two patients withdrew from the study for social rather than medical reasons. Therefore, data were analysed for the remaining thirty patients. Three patients required major reductions in dosage because of CYP2D6 poor metabolizer phenotype [43]. Median plasma Px concentration after two weeks’ treatment was 0.26 (0.25, 0.43) mg/L (therapeutic range 0.15–0.6 mg/L) [15,44]. 

Table 2 summarizes effects of Px on parameters of insulin secretion and of tissue responsivenss to insulin. HOMA-IR score, the primary endpoint of the study, increased significantly post Px treatment, indicating accentuation of insulin resistance. This change reflected an approximately 13% increase in plasma insulin concentrations, without significant change in fasting blood glucose levels. No patient experienced any symptomatic hypoglycaemic episodes. 

**Figure 1:**
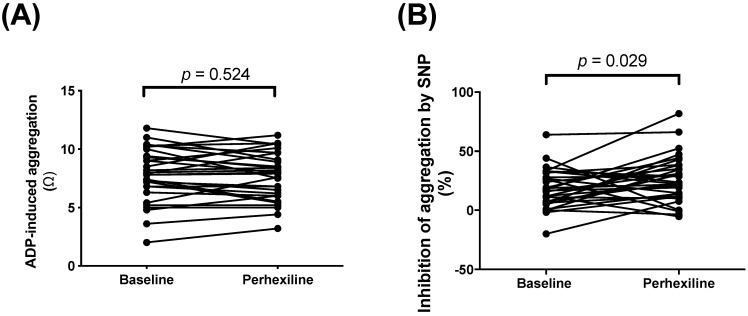
Effects of Px therapy on (ADP-induced platelet aggregation (**A**), and inhibition of ADP-induced platelet aggregation by sodium nitroprusside (SNP) (**B**).

As previously reported [14], Px therapy did not significantly affect extent of ADP-induced platelet aggregation (Figure 1A), but potentiated anti-aggregatory effects of the NO donor SNP from 17.6 ± 3.0 to 26.4 ± 3.7% (*p* = 0.029, Figure 1B). 

**Figure 2:**
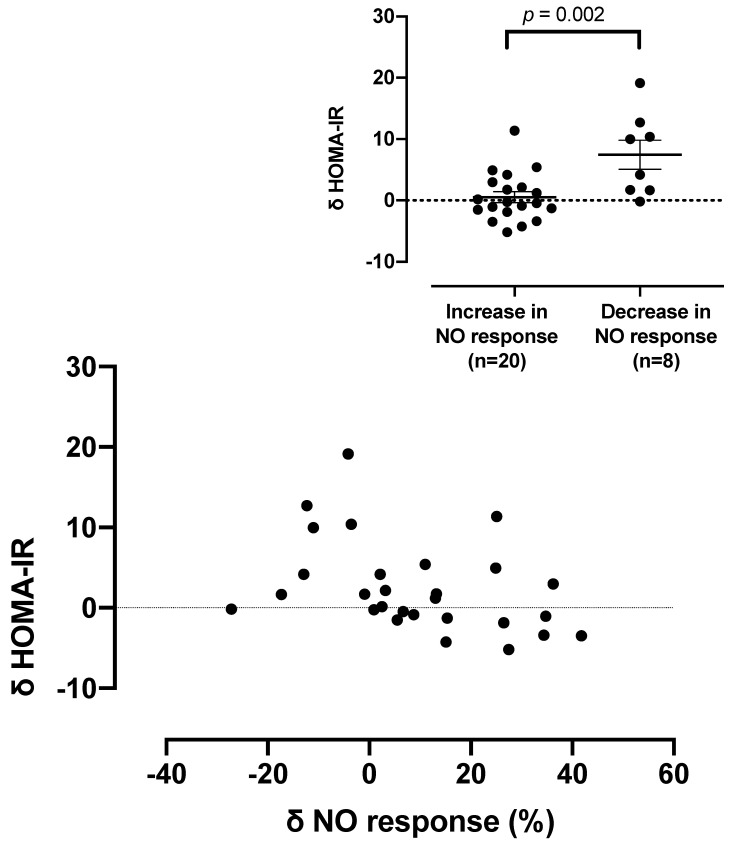
Correlation between changes in platelet responses to nitric oxide and in insulin sensitivity in patients treated with Px: r = −0.304, *p* = 0.1.

To determine whether changes in HOMA-IR values in individual patients also related to increases in NO sensitivity induced by Px, correlations were sought between proportional change in HOMA-IR and in SNP response in individual patients. The results, shown in Figure 2, indicated that despite the overall increase in HOMA-IR induced by Px, sensitization to NO tended to be associated with decreases in HOMA-IR. This non-signficant relationship reached significance (r = −0.40, *p* = 0.037) if outlying datapoints were removed. Furthermore, when these data were analysed categorically, according to whether or not there was any increase in sensitization of NO (Figure 2 inset), increases in HOMA-IR were substantially greater (*p* = 0.002) in patients without any sensitization to NO. 

**Figure 3:**
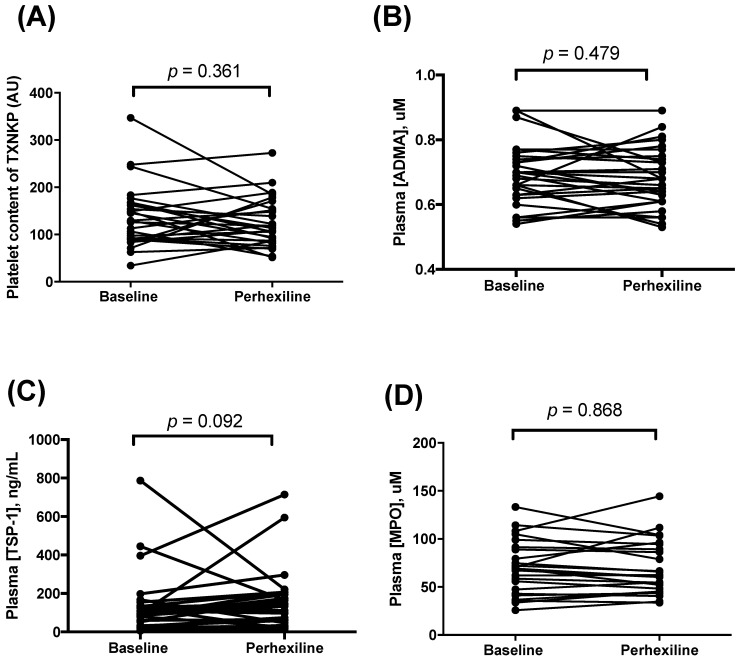
The effect of Px on platelet content of TXNIP (**A**), plasma concentrations of ADMA (**B**), TSP-1 (**C**) and MPO (**D**).

Platelet content of TXNIP and plasma concentrations of ADMA, MPO and TSP-1 did not change significantly under treatment with Px (Figure 3). Furthermore, there was no significant relationship between individual patient fluctuations in TXNIP expression changes in NO responses, and changes in HOMA-IR (data not shown). 

## 4. Discussion

Px has long been established as a potent prophylactic anti-anginal agent, whether used as monotherapy or in combination with other drugs [44,45,46]. It has been shown that Px improves symptomatic status and left ventricular systolic function in patients with systolic heart failure [21], as well as cardiac energetics in patients with dilated and hypertrophic cardiomyopathy [20,22]. Finally, recent preclinical studies have established the potential utility of Px in the treatment of malignancies, both as a sensitizer to chemotherapy or as a tumour-suppressive agent [24,25,26,27]. Therefore, in theory Px represents an agent with twin advantages: tumour suppression and simultaneous cardioprotection in the face of potentially cardiotoxic therapies. Table 3 summarizes our current understanding of the biochemical actions, clinical utility and potentials for its future clinical use of Px. 

The main theoretical barrier to the widespread use of Px in the treatment both of cardiovascular disease and of malignancy is therefore the potential for induction of hepato- and neurotoxicity. However, the potential for Px to induce hepatitis and/or peripheral neuropathy during long-term therapy has been dramatically reduced by the availability of therapeutic drug monitoring of plasma concentrations of Px and of its hydroxylated metabolites [16,47,48]. Thus, the only remaining concern is the risk of hypoglycaemia, which has been reported as a rare but potentially serious adverse effect in some case reports [29,30,49], even though the cause of hypoglycaemia remained uncertain. Therefore, the primary objective of the current study was to determine whether induction of hypoglycaemia remains a significant problem when Px is utilized for treatment of heart disease in patients with diabetes.

The results of the study indicate that short-term Px therapy, titrated to achieve therapeutic plasma Px concentrations, does not affect fasting blood glucose levels, while significantly increasing plasma insulin concentrations. On this basis, Px technically increased insulin resistance, as measured by HOMA-IR. Furthermore, consistent with previous observations in patients with severe angina pectoris, Px normalizes anti-aggregatory responses to the NO donor SNP, and thus ameliorates “NO resistance”, a condition known to be an independent negative prognostic marker [5,6]. This is an important finding, especially in patients with diabetes, as they are at increased risk of adverse outcomes in the presence of acute myocardial ischaemia or heart failure [50]. 

To test the hypothesis that the impact of Px on HOMA-IR and platelet responsiveness to NO reflects a common mechanistic pathway, we sought evidence of correlation between these parameters. While there was no significant relationship (without removal of outlying datapoints), the two parameters tended to have an inverse correlation. When data were compared in a categorical manner (Figure 3), increases in HOMA-IR were substantially greater in patients in whom no sensitization to NO occurred. Therefore, consistent with our previous finding that insulin infusion administered to patients to correct hyperglycaemia also reverses NO resistance [7], it is likely that increased insulin effect occurs in some Px-treated patients with similar outcomes. We have also previously shown that in patients with polycystic ovarian syndrome, platelet responsiveness to NO is a significant multivariate correlate of insulin responsiveness [51], suggesting that the relationship shown in Figure 3 was driven by sensitization to NO, irrespective of its induction in this case by Px. 

As originally proposed by Randle et al. [19], fatty acids and glucose compete for selection and oxidation by muscles and adipose tissues. Therefore, inhibition of fatty acid metabolism induces a shift towards glucose utilization, potentially mediating increases in cardiac metabolic efficiency. If glucose utilization were increased simultaneously with glucose uptake into tissues such as muscle, this could potentially induce hypoglycaemia. However, in many circumstances, especially during the fed state, insulin effects on tissue uptake of glucose are primarily associated with increased glycogen synthesis, rather than glucose utilization [52]. Therefore, increased plasma concentrations of insulin in the presence of Px do not always imply increased oxidation of glucose: it may well be that insulin secretion is not in any way a mediator of the “Randle cycle”. Indeed, previous studies have suggested a dissociation of insulin signaling from substrate utilization [19,53]. 

The mechanism(s) for increases in plasma insulin concentrations are uncertain. Px may increase plasma insulin concentrations potentially through CPT-1 inhibition at the pancreatic islet beta-cells. It was previously demonstrated that the sulphonylurea glibenclamide inhibited CPT-1 in islet cells in a K_ATP_-independent manner, as did another CPT-1 inhibitor, etomoxir, thereby stimulating the exocytosis of insulin [54]. Px may well exert a similar effect. If so, the observed increase in plasma insulin levels might result from insulin exocytosis rather than a failure of intracellular effect. 

The study has some limitations. First, it is entirely possible, given the results, that risk of hypoglycaemia with Px may be greater in non-diabetic patients, given integrity of glucose uptake mechanisms, but this remains to be explored. We also do not know whether hyperinsulinaemia as a driver of insulin resistance carries adverse prognostic implications in the long-term, given that the prognostic implications of insulin hypersecretion are controversial [55]. A larger sample size with longer duration of investigations would be necessary to evaluate this possibility, and also to explore the prognostic implications of heterogenous Px effect on insulin secretion versus responsiveness to NO. Finally, we do not yet understand the extent to which these findings are relevant to the emerging role of Px as an antineoplatic agent, but would emphasise that (1) in this circumstance, the dependency of many cancers on CPT-modulated fatty acid uptake is likely to be a key mechanism of Px action, and (2) that cancer occurs particularly frequently in diabetes [56] and the current results suggest that Px represents a safe modality of treatment in such individuals. 

## 5. Conclusions

In conclusion, in patients with stable T2D, short-term treatment with Px does not induce changes in fasting blood glucose levels, increases plasma insulin concentrations and sensitizes platelets to the anti-aggregatory effects of NO. The latter two effects are potentially, but not definitely, inter-related. 

## Figures and Tables

**Table 1:** Baseline clinical characteristics (n = 30).

Patient Characteristics	
Age (years)	70 ± 2.2
Female (%)	37
HbA1c (%)	7.1 ± 0.21
Baseline serum creatinine (μmol/L)	112.7 ± 13.74
**Major indication(s) for Px therapy**	
Refractory angina (%)	70
Systolic heart failure (%)	23
Symptomatic aortic stenosis (%)	7
**Concurrent pharmacotherapy**	
ACE-inhibitor/ARB (%)	73
Calcium channel antagonist (%)	43
β-blocker (%)	37
Organic nitrate (%)	40
Metformin (%)	60
Insulin (%)	33
Sulphonylurea (%)	30
DPP-IV inhibitor or thiazolidinedione (%)	20

HbA1c = glycosylated haemoglobin; ARB = angiotensin receptor blocker; DPP-IV = dipeptidyl peptidase-IV.

**Table 2:** Effects of two weeks of Px therapy on blood glucose, plasma insulin levels and insulin sensitivity scores. HOMA-IR (homeostatic model assessment of insulin resistance, derived from product of plasma insulin and glucose concentrations; fasting insulin (microU/L) × fasting glucose (nmol/L)/22.5) and QUICKI (derived from reciprocal of log fasting insulin plus log fasting glucose concentrations; 1/[log(fasting insulin microU/mL) + log(fasting glucose mg/dL)]).

Parameter	Before Px	After Px	*p* Value
Fasting blood glucose level (mmol/L)	6.8 (5.7, 8.6)	7.0 (6.0, 8.7)	0.366
Fasting plasma insulin level (mU/L)	16.5 (11.8, 25.3)	19.0 (11.8, 37.3)	0.014
HOMA-IR score	4.47 (3.42, 8.55)	6.15 (3.05, 15.06)	0.028
QUICKI score	0.158 ± 0.001	0.156 ± 0.002	0.078

**Table 3:** Perhexiline: current “State of the art” regarding its biochemical actions, utility & toxicity.

Effects	Toxicity
Inhibition of CPT-1 Improvement in cardiac energetics, potentially via “Randle shift” Potentiation of anti-aggregatory effects of nitric oxide	Potential for phospholipid accumulation in liver and nerves
**Known utility**	
Prophylaxis of exertional angina Accessory therapy for systolic heart failure: improved symptomatic status	Short-term nausea, dizziness or occasional hypoglycaemia Long-term hepatitis and peripheral neuropathy, subject to elevation of plasma Px concentrations
**Potential for incremental use**	
Limitation of symptoms in hypertrophic cardiomyopathy	
Perioperative therapy in patients with severe aortic stenosis Ancillary therapy in cancer, as cardioprotective agent during chemotherapy and/or as chemotherapy	

## Data Availability

Request to original data should be addressed to the corresponding author and may be made available according to local procedures.
